# Risk factors analysis of hypokalemia after radical resection of esophageal cancer and establishment of a nomogram risk prediction model

**DOI:** 10.3389/fsurg.2024.1433751

**Published:** 2025-01-07

**Authors:** Guanqiang Yan, Jingxiao Li, Yiji Su, Guosheng Li, Guiyu Feng, Jun Liu, Xiang Gao, Huafu Zhou

**Affiliations:** Department of Cardio-Thoracic Surgery, The First Affiliated Hospital of Guangxi Medical University, Nanning, Guangxi, China

**Keywords:** esophageal cancer, radical resection of esophagus cancer, hypokalemia, prediction model, nomogram

## Abstract

**Objective:**

This study aimed to explore the risk factors of hypokalemia after radical resection of esophageal cancer (EC) and establish a nomogram risk prediction model to evaluate hypokalemia risk after esophagectomy. Thus, this study provides a reference for the clinical development of intervention measures.

**Methods:**

Clinical data of EC patients who underwent radical surgery from January 2020 to November 2022 in the First Affiliated Hospital of Guangxi Medical University were retrospectively collected. The relevant variables were screened using multivariate logistic regression analysis with IBM SPSS 25.0 and R 4.2.0 software, and a nomogram for predicting hypokalemia risk was established. The established nomogram was evaluated by receiver operating characteristic (ROC), calibration, and decision curves. The model was also internally validated by 1000 bootstrap resampling methods.

**Results:**

After radical EC resection, the incidence rate of hypokalemia in 213 patients was 19.2% (41/213). The hemoglobin levels, total serum protein, serum albumin, calcium ion concentration, direct bilirubin, prothrombin time (PT), and activated partial thromboplastin time (APTT) were related (*p* < 0.05). The multivariate logistic analysis showed that the white blood cell count, serum albumin level, direct bilirubin, and operation time were risk factors for hypokalemia after radical EC resection (*p* < 0.05). The area under the ROC curve (AUC) was 0.764, demonstrating the good discriminative ability of the established nomogram for hypokalemia prediction. The calibration curve showed a good fit between the predicted and actual observed probabilities. The model maintained a high C-index in the internal validation (C-index = 0.758), supporting that the nomogram can be widely used for hypokalemia prediction.

**Conclusion:**

The prediction model for hypokalemia risk with individualized scores based on the patient's white blood cell count, serum albumin level, direct bilirubin, and operation time can screen out high-risk patients who might develop hypokalemia. It is of certain reference value for clinicians to screen and follow up with patients with emphasis and to formulate preoperative and postoperative intervention strategies.

## 1 Introduction

Esophageal cancer (EC) is one of the most common digestive tract malignancies (1). With the development of new treatment methods and early prediction techniques, the five-year survival rate of EC patients without metastasis can reach 35% (2). Endoscopic and open surgery is still one of the effective treatments for EC in the early stages, but these methods often lead to serious complications (3). Hypokalemia refers to a lower concentration of potassium ions than the normal level in the blood, often causing symptoms such as muscle strength decline, arrhythmia, and syncope (4). Hypokalemia is a common electrolyte metabolic disorder mainly diagnosed by testing blood samples from patients. However, laboratory tests also have limitations: before blood samples are sent for postoperative examination, some patients with hypokalemia have discomfort, leading to a delayed diagnosis (5). On the other hand, patients are in an extremely weak state postoperatively and repeated blood tests to determine electrolyte imbalance might aggravate their condition (6). Additionally, hypokalemia is a common but extremely complex issue. It often requires drugs, electrolyte drinks, and other treatments. Besides, the treatment cycle is very long, and the economic burden on patients is relatively large.

In a randomized controlled study, Zhang et al. showed that the incidence of postoperative hypokalemia in EC patients is as high as 20% despite multiple treatment modalities (7). Therefore, if hypokalemia can be predicted using other blood indicators sent for postoperative examination, the medical costs would reduce, and patient prognosis would improve under the premise of controlling the disease. In the past, some scholars have reported scoring systems for assessing surgical risk, including the POSSUM score and the E-PASS scoring system (8, 9). However, these scoring systems and their variables remain controversial. Moreover, the hypokalemia risk after esophagectomy cannot be quantified, which hinders risk-stratified interventions for patients. Additionally, the lack of recognized risk factors with a decisive impact on hypokalemia limits the establishment of reliable predictive models and standardized risk assessment tools to some extent. Therefore, exploring the risk factors and establishing a prediction model for the hypokalemia risk after EC resection based on individualized scores are crucial.

Therefore, this study analyzed the clinical data of 213 patients who underwent esophagectomy to explore the potential risk factors of postoperative hypokalemia in EC patients and construct a model to predict hypokalemia risk.

## 2 Materials and methods

### 2.1 General information

**Figure 1:**
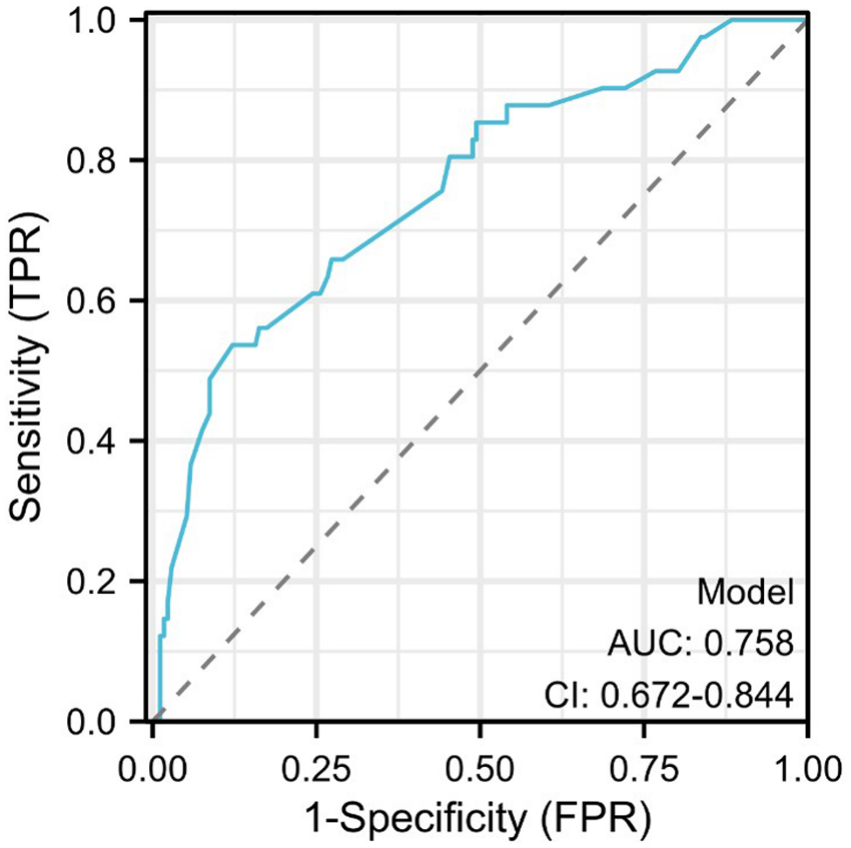
Schematic diagram of patient screening process.

A total of 213 patients who underwent radical resection of esophageal cancer in the Thoracic Surgery Department of the First Affiliated Hospital of Guangxi Medical University from January 2020 to November 2022 were selected as the research subjects. Detailed patient screening process was shown in Figure 1. The inclusion criteria were: (1) Primary esophageal cancer diagnosed by electronic gastroscopic biopsy and underwent radical EC resection; (2) No other surgery was combined during the operation; (3) Hypokalemia was not diagnosed before the operation. The exclusion criteria were: (1) Hypokalemia diagnosed before the operation; (2) Non-esophageal surgery was combined during the operation; (4) Organ dysfunction in the preoperative examination; (5) Neoadjuvant chemotherapy or radiotherapy; (6) The surgical method was changed during the operation; (7) Surgical accident occurred during the operation; (8) Non-hypokalemia-related death occurred after the operation. The Ethics Committee of the First Affiliated Hospital of Guangxi Medical University approved this retrospective study.

### 2.2 Clinical data

General and perioperative clinical data of patients were collected and analyzed using the clinical medical record system. The general clinical data included gender, age, body mass index (BMI), hypertension, diabetes, coronary heart disease, smoking history, and drinking history. The perioperative clinical data included the number of red blood cells, white blood cells, and platelets on the first day after surgery, serum albumin level, electrolytes, total bilirubin, direct bilirubin, alanine aminotransferase (ALT), aspartate aminotransferase (AST), serum creatinine, thromboplastin time (PT), activated partial thromboplastin time (APTT), operation method, and operation time. The hematological data were obtained from the laboratory tests conducted in the First Affiliated Hospital of Guangxi Medical University within one day after the operation to reduce the research error. The pathological staging of all patients was based on tumor size, invasion depth, number of positive lymph nodes, and distant metastasis (TNM), referring to the eighth edition of the American Joint Cancer Society (AJCC) Tumor Staging Manual (10). This study complied with the Declaration of Helsinki; all patients signed the informed consent form.

### 2.3 Diagnostic criteria for hypokalemia

The criteria for diagnosing hypokalemia by electrocardiogram were ST-segment depression >0.05 mV, T-wave bidirectional inversion, U-wave increase >0.1 mV, and arrhythmia. The standard for diagnosing hypokalemia with a biochemical examination was a serum potassium concentration under 3.5 mmol/L.

### 2.4 Establishment and interpretation of the nomogram risk model

Multivariate logistic analysis was used to screen out risk factors significantly related to postoperative hypokalemia in esophageal cancer. Through the analysis of a large amount of clinical data, appropriate statistical tests (such as *t*-test, analysis of variance, etc.) were used for continuous variables, and chi-square test was used for categorical variables. After determining the risk factors, the regression coefficients of each factor were obtained through regression analysis and used as weights to construct a risk prediction model. Specifically, statistically significant risk factors were incorporated into the nomogram, and corresponding scores were assigned to each factor. The determination of scores is reasonably transformed according to the magnitude of regression coefficients, so that clinicians can intuitively calculate the total score according to the various indicators of patients, and then evaluate the risk probability of patients developing a specific outcome.

### 2.5 Risk prediction model evaluation

To evaluate the validity and reliability of the model, we computed the concordance index (C-index) as part of the logistic regression analysis. Additionally, we generated a receiver operating characteristic curve (ROC) for the model. The area under the ROC curve (AUC) indicates the predictive ability of the model, with a score approaching 1 signifying stronger predictive power. This interpretation aligns with the significance of the C-index. Furthermore, we constructed a calibration curve to assess the goodness-of-fit of the model by examining the degree of fit between predicted and actual probabilities. Finally, we utilized decision curve analysis (DCA) to evaluate the utility of various risk factors.

### 2.6 Statistical methods

IBM SPSS 25.0 software was used for statistical analyses. Measurement data are expressed as means ± standard deviations (*x* ± s), and groups were compared by independent sample *t*-test. Count data are expressed as percentages, and the *χ*^2^ test was used for comparisons. According to previous studies, the reference value range was grouped by age, sex, body mass index (BMI), and blood test results. The receiver operating characteristic (ROC) curve and the Youden index were used to determine the best critical value for operation time. Multivariate logistic analysis was used to analyze the risk factors of postoperative hypokalemia (Stepwise method). The “rms,” “proc,” and “hmisc” R packages were used to incorporate the risk factors into the nomogram based on the multivariate logistic analysis to establish the risk prediction model. Bootstrap (BH = 1,000) was used for internal verification, a diagnostic calibration curve was constructed, and the consistency index (C-index) was calculated. C-index = 0.50 means that the model has no predictive effect, 0.50 < C-index ≤ 0.70 indicates poor model predictive value, 0.70 < C-index ≤ 0.90 indicates that the model prediction value is good, 0.90 < C-index < 1 indicates that the model prediction value is excellent, and C-index = 1 indicates that the model prediction is completely consistent with the actual value. A *p* < 0.05 was considered statistically significant.

## 3 Results

### 3.1 Characteristics of the study population

A total of 213 eligible patients with esophageal cancer were included in this study. Among them, 178 were males, 35 were females, 62 were ≥65 years old, and 151 were <65 years old. Regarding TNM stages, 48 cases were stage I, 87 were stage II, 73 were stage III, and five were stage IV. Patients were divided into hypokalemia (serum *K*^+^ < 3.5 mmol/L) with 41 cases (19.2%) and non-hypokalemia groups according to the electrolyte results within one day after the operation. The blood group (serum *K*^+^ ≥ 3.5 mmol/L) included 172 cases (80.8%), 178 males (83.6%), and 35 females (16.4%), with an average age of 59.31 ± 9.37 years and an average BMI of 21.48 ± 3.12. Patient demographics and general clinical characteristics are shown in Table 1. In the hypokalemia group, the frequency of patients receiving minimally invasive surgery was 39.13% and open surgery was 13.77%, which indicated that hypokalemia risk is higher in patients undergoing thoracoscopic assisted radical EC resection than open surgery (*p* < 0.001). Nevertheless, the drinking history, hypertension history, diabetes history, tumor TNM stage, G stage, and clinical stage did not differ.

**Table 1:** Baseline characteristics of patients after radical resection of esophageal cancer.

Clinical indicators	Hypokalemia (*n* = 41)	no hypokalemia (*n* = 172)	Total (*n* = 213)	*p*
Gender				
Male	31 (75.6)	147 (85.5)	178 (83.6)	0.195
Female	10 (24.4)	25 (14.5)	35 (16.4)	
Age (year)				
>=65	16 (39.0)	46 (26.7)	62 (29.1)	0.173
<65	25 (61.0)	126 (73.3)	151 (70.9)	
Smoking_History				
No	23 (56.1)	69 (40.1)	92 (43.2)	0.093
Yes	18 (43.9)	103 (59.9)	121 (56.8)	
Drinking_History				
No	21 (51.2)	68 (39.5)	89 (41.8)	0.235
Yes	20 (48.8)	104 (60.5)	124 (58.2)	
Hypertension				
No	34 (82.9)	138 (80.2)	172 (80.8)	0.863
Yes	7 (17.1)	34 (19.8)	41 (19.2)	
Diabetes				
No	41 (100.0)	162 (94.2)	203 (95.3)	0.242
Yes	0 ( 0.0)	10 ( 5.8)	10 (4.7)	
Coronary_Heart_disease				
No	33 (80.5)	155 (90.1)	188 (88.3)	0.147
Yes	8 (19.5)	17 ( 9.9)	25 (11.7)	
Surgical_Method (%)				
Minimal invasion	18 (43.9)	28 (16.3)	46 (21.6)	**<0** **.** **001**
Open	23 (56.1)	144 (83.7)	167 (78.4)	
T (%)				
T1	8 (19.5)	34 (19.8)	42 (19.7)	0.951
T2	8 (19.5)	38 (22.1)	46 (21.6)	
T3	23 (56.1)	89 (51.7)	112 (52.6)	
T4	2 (4.9)	11 (6.4)	13 (6.1)	
N (%)				
N0	24 (58.5)	99 (57.6)	123 (57.7)	0.599
N1	12 (29.3)	42 (24.4)	54 (25.4)	
N2	5 (12.2)	25 (14.5)	30 (14.1)	
N3	0 (0.0)	6 (3.5)	6 (2.8)	
M (%)				
M0	41 (100.0)	170 (98.8)	211 (99.1)	0.786
M1	0 (0.0)	1 (0.6)	1 (0.5)	
M2	0 (0.0)	1 (0.6)	1 (0.5)	
G (%)				
G1	11 (26.8)	57 (33.1)	68 (31.9)	0.488
G2	27 (65.9)	96 (55.8)	123 (57.7)	
G3	3 (7.3)	19 (11.0)	22 (10.3)	
Stage (%)				
I	7 (17.1)	41 (23.8)	48 (22.5)	0.36
II	20 (48.8)	67 (39.0)	87 (40.8)	
III	12 (29.3)	61 (35.5)	73 (34.3)	
IV	2 (4.9)	3 (1.7)	5 (2.3)	

The differences in laboratory test results between the hypokalemia and non-hypokalemia groups were further compared (Table 2). Compared to the non-hypokalemia group, the red blood cell count, hemoglobin level, total serum protein, serum albumin, and calcium ion concentration were lower (*p* < 0.05), while direct bilirubin, PT, and APTT were higher (*p* < 0.05) and the operation time was longer (*p* < 0.001) in the hypokalemia group. The two groups also differed for BMI, white blood cell count, platelets, the absolute value of neutrophils, creatinine, and endogenous creatinine clearance rate but without statistical significance (*p* > 0.05). The 22 baseline features were reduced to eight potential predictors, and the *χ*^2^ test suggested that the strongest predictors were white blood cell count, platelets, serum albumin, calcium ions, total bilirubin, direct bilirubin erythrocytes, PT, and operation time (Table 3).

**Table 2:** Analysis of clinical data of patients undergoing radical resection of esophagus cancer.

	Hypokalemia (*n* = 41)	No Hypokalemia (*n* = 172)	Overall (*n* = 213)	*p*
Age	61.80 ± 8.16	58.71 ± 9.56	59.31 ± 9.37	0.057
BMI	22.10 ± 3.75)	21.34 ± 2.94)	21.48 ± 3.12	0.157
White blood cell	12.88 ± 5.33	13.30 ± 4.45	13.22 ± 4.62	0.601
Red blood cell	3.33 ± 0.73	3.69 ± 0.97	3.63 ± 0.94	**0** **.** **026**
Platelet	229.31 ± 131.89	250.33 ± 110.86	246.28 ± 115.17	0.295
Hemoglobin	97.50 ± 18.57	106.60 ± 17.75	104.85 ± 18.22	**0**.**004**
Absolute value of neutrophil	11.07 ± 4.38	11.53 ± 4.28	11.45 ± 4.29	0.538
Absolute value of lymphocyte	0.93 ± 0.51	1.04 ± 1.44	1.02 ± 1.32	0.627
Total protein	55.00 ± 9.29	57.81 ± 6.20	57.27 ± 6.96	**0**.**02**
ALB	31.74 ± 4.27	33.94 ± 4.28	33.51 ± 4.36	**0**.**003**
Serum-Na^+^	137.74 ± 5.53	137.82 ± 3.39	137.80 ± 3.87	0.914
Serum-Ca2^+^	1.87 ± 0.39	1.99 ± 0.32	1.97 ± 0.33	**0**.**047**
Total bilirubin	16.03 ± 8.64	15.29 ± 12.42	15.43 ± 11.77	0.718
Direct bilirubin	7.46 ± 6.71	5.09 ± 3.88	5.55 ± 4.64	**0**.**003**
Indirect bilirubin	9.20 ± 4.59	10.17 ± 11.02	9.99 ± 10.10	0.581
AST	47.71 ± 35.57	43.80 ± 23.75	44.55 ± 26.38	0.396
ALT	32.12 ± 32.51	28.66 ± 26.27	29.32 ± 27.53	0.47
Cr	67.34 ± 34.91	73.07 ± 22.69	71.97 ± 25.50	0.197
Endogenous creatinine clearance	97.28 ± 34.43	98.06 ± 27.37	97.91 ± 28.77	0.876
PT	13.71 ± 2.17	12.74 ± 1.39	12.93 ± 1.61	**<0**.**001**
APTT	32.19 ± 7.67	30.38 ± 3.21	30.73 ± 4.47	**0**.**02**
Duration of surgery	320.83 ± 163.66	227.07 ± 102.01	245.11 ± 121.74	**<0**.**001**

**Table 3:** Potential predictors clinical features for initial screening.

Characteristics	No Hypokalemia (*n* = 172)	Hypokalemia (*n* = 41)	*P* value
White blood cell, *n* (%)			**0** **.** **025**
Abnormal	153 (71.8%)	31 (14.6%)	
Normal	19 (8.9%)	10 (4.7%)	
Platelet, *n* (%)			0.080
Normal	132 (62%)	26 (12.2%)	
Abnormal	40 (18.8%)	15 (7%)	
ALB, *n* (%)			**0**.**023**
Normal	66 (31%)	8 (3.8%)	
Abnormal	106 (49.8%)	33 (15.5%)	
Serum-Ca2^+^, *n* (%)			0.132
Abnormal	118 (55.4%)	33 (15.5%)	
Normal	54 (25.4%)	8 (3.8%)	
Total bilirubin, *n* (%)			0.142
Normal	143 (67.1%)	30 (14.1%)	
Abnormal	29 (13.6%)	11 (5.2%)	
Direct bilirubin, *n* (%)			**0**.**002**
Normal	143 (67.1%)	25 (11.7%)	
Abnormal	29 (13.6%)	16 (7.5%)	
PT, *n* (%)			0.152
Normal	158 (74.2%)	34 (16%)	
Abnormal	14 (6.6%)	7 (3.3%)	
Duration of surgery, *n* (%)			**0**.**004**
Normal	61 (28.6%)	5 (2.3%)	
Abnormal	111 (52.1%)	36 16.9%)	

(1) White blood cell (10^9^/L): abnormal <3.5 or >9.5, normal 3.5–9.5, *p* = 0.025 indicates a statistically significant difference between the two groups for abnormal white blood cell count; (2) Platelet (10^9^/L): abnormal <125 or >350, normal 125–350; (3) ALB (g/L): abnormal <35, normal >= 35, *p* = 0.023 indicates a statistically significant difference for abnormal albumin levels; (4) Serum-Ca2^+^ (mmol/L): abnormal <2.52 or >2.11, normal 2.11–2.52; (5) Total bilirubin (*μ*mol/L): abnormal <3.4 or >20.5, normal 3.4–20.5; (6) Direct bilirubin (μmol/L): abnormal >6.8, normal 0–6.8, *p* = 0.002 indicates a statistically significant difference for abnormal direct bilirubin levels; (7) PT(s) < 9 or >15, normal 9–15; (8) Duration of surgery(min): abnormal >=180, normal <180, *p* = 0.004 indicates a statistically significant difference for longer surgery duration.

### 3.2 Risk factors for hypokalemia

The multivariate logistic analysis showed that the white blood cell count, serum albumin level, direct bilirubin, and operation time were independent risk factors for hypokalemia in patients after radical EC resection (*p* < 0.05) (Table 4).

**Table 4:** Uni- and multivariate logistic regression analyses.

Characteristics	Total (*N*)	Univariate analysis		Multivariate analysis	
		Odds Ratio (95% CI)	*P* value	Odds Ratio (95% CI)	*P* value
White blood cell	213				
Abnormal	184	Reference		Reference	
Normal	29	2.598 (1.102–6.123)	**0** **.** **029**	3.795 (1.433–10.054)	**0**.**007**
Platelet	213				
Normal	158	Reference		Reference	
Abnormal	55	1.904 (0.920–3.940)	0.083	1.460 (0.653–3.266)	0.357
ALB	213				
Normal	74	Reference		Reference	
Abnormal	139	2.568 (1.119–5.898)	**0**.**026**	2.490 (1.001–6.193)	0.050
Serum-Ca2+	213				
Abnormal	151	Reference			
Normal	62	0.530 (0.229–1.223)	0.137		
Total bilirubin	213				
Normal	173	Reference			
Abnormal	40	1.808 (0.814–4.015)	0.146		
Direct bilirubin	213				
Normal	168	Reference		Reference	
Abnormal	45	3.156 (1.500–6.639)	**0**.**002**	2.964 (1.304–6.738)	**0**.**009**
PT	213				
Normal	192	Reference		Reference	
Abnormal	21	2.324 (0.872–6.191)	0.092	1.752 (0.547–5.614)	0.345
Duration of surgery	213				
Normal	66	Reference		Reference	
Abnormal	147	3.957 (1.476–10.608)	**0**.**006**	3.233 (1.166–8.964)	**0**.**024**

### 3.3 Nomogram risk prediction model for hypokalemia patients after radical EC resection

**Figure 2:**
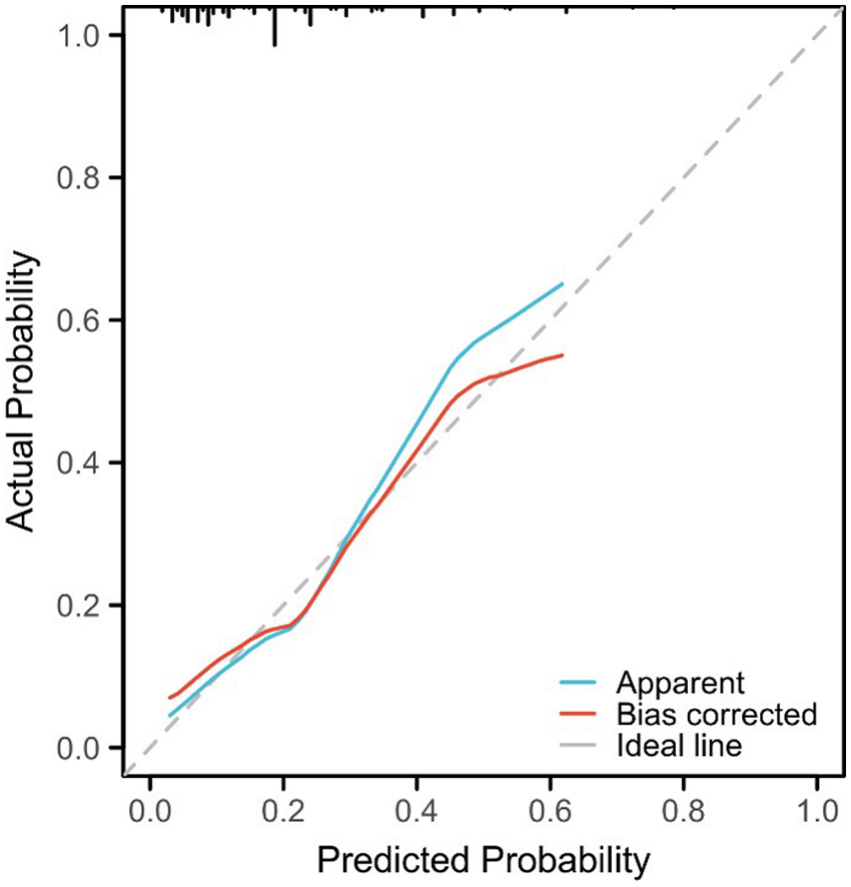
Nomogram constructed based on the independent risk factors.

**Figure 3:**
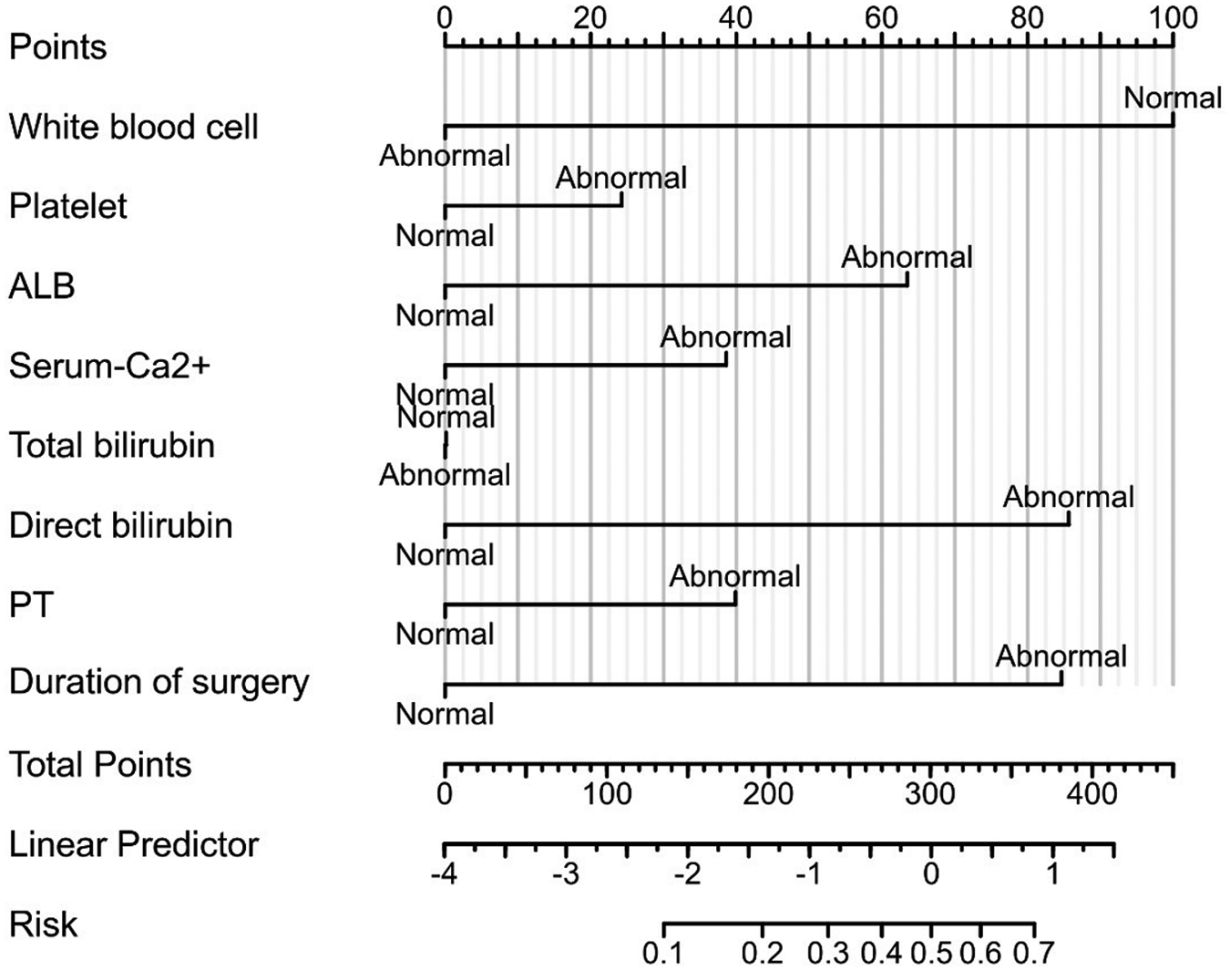
Receiver operating characteristic (ROC) curve.

The risk factors based on multivariate Logistic analysis were included in the nomogram to establish a risk prediction model for hypokalemia patients after radical EC resection. Each risk factor was scored separately. White blood cell count >3.5 × 10^9^/L and <9.5 × 10^9^/L was considered “Normal” and rated as 100 points, and ≥9.5 × 10^9^/L was “Abnormal” and rated as 0 points; serum albumin level ≥35 g/L was considered “Normal” and rated as 0 points, and <35 g/L was “Abnormal” and rated as 66 points; direct bilirubin ≤6.8 *μ*mol/L was considered “Normal” and rated as 0 points, and >6.8 *μ*mol/L was “Abnormal” and rated as 90 points; operation time ≤180 min was regarded as “Normal” and rated as 0 points, >180 min was “Abnormal” and rated as 90 points. These factors were chosen for their significant association with the risk of postoperative hypokalemia. For example, a patient with a white blood cell count of 10 × 10^9^/L, serum albumin of 30 g/L, direct bilirubin of 7.5 *μ*mol/L, and an operation time of 200 min would score 246 points (0 + 66 + 90 + 90). Based on the nomogram, this total corresponds to an estimated risk of hypokalemia of approximately 45%. Higher scores reflected greater risk: elevated white blood cell count, low serum albumin, high direct bilirubin, and prolonged operation time all increased the likelihood of hypokalemia. The total score was the sum of individual scores and corresponded to the risk of postoperative hypokalemia (130 points correspond to a probability of 0.1, and 370 points correspond to 0.7) (Figure 2). The area under the ROC curve (AUC) of the model was 0.758, demonstrating its good discriminant ability for hypokalemia prediction (Figure 3).

### 3.4 Validation of the accuracy of the nomogram risk prediction model

**Figure 4:**
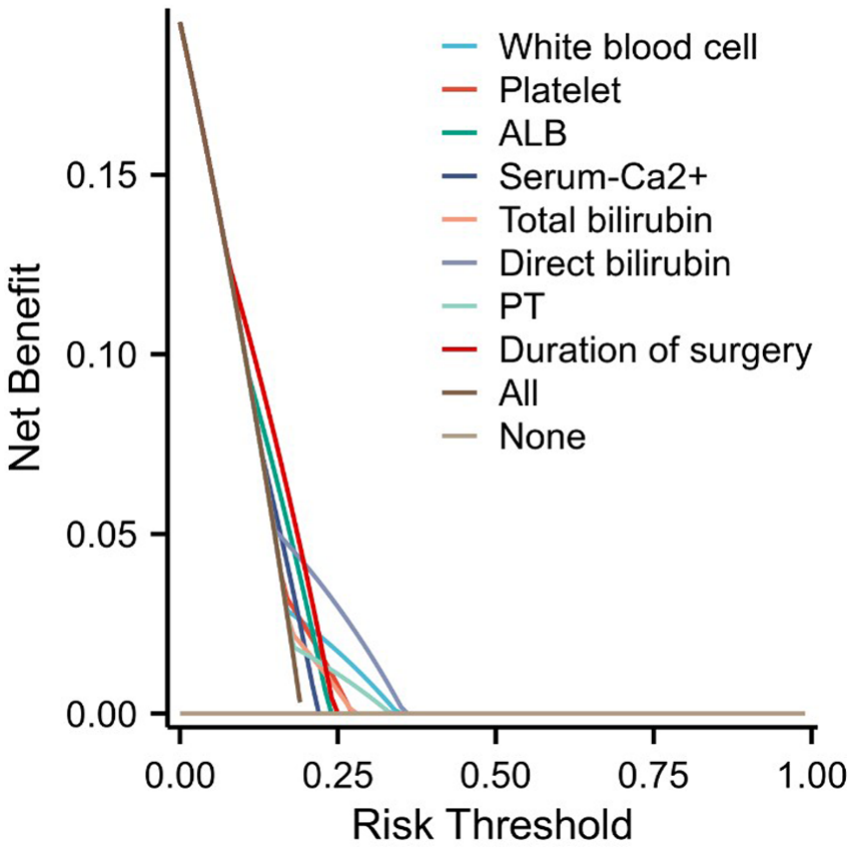
Calibration plot (by a Bootstrap method with 1,000 resamples).

**Figure 5:**
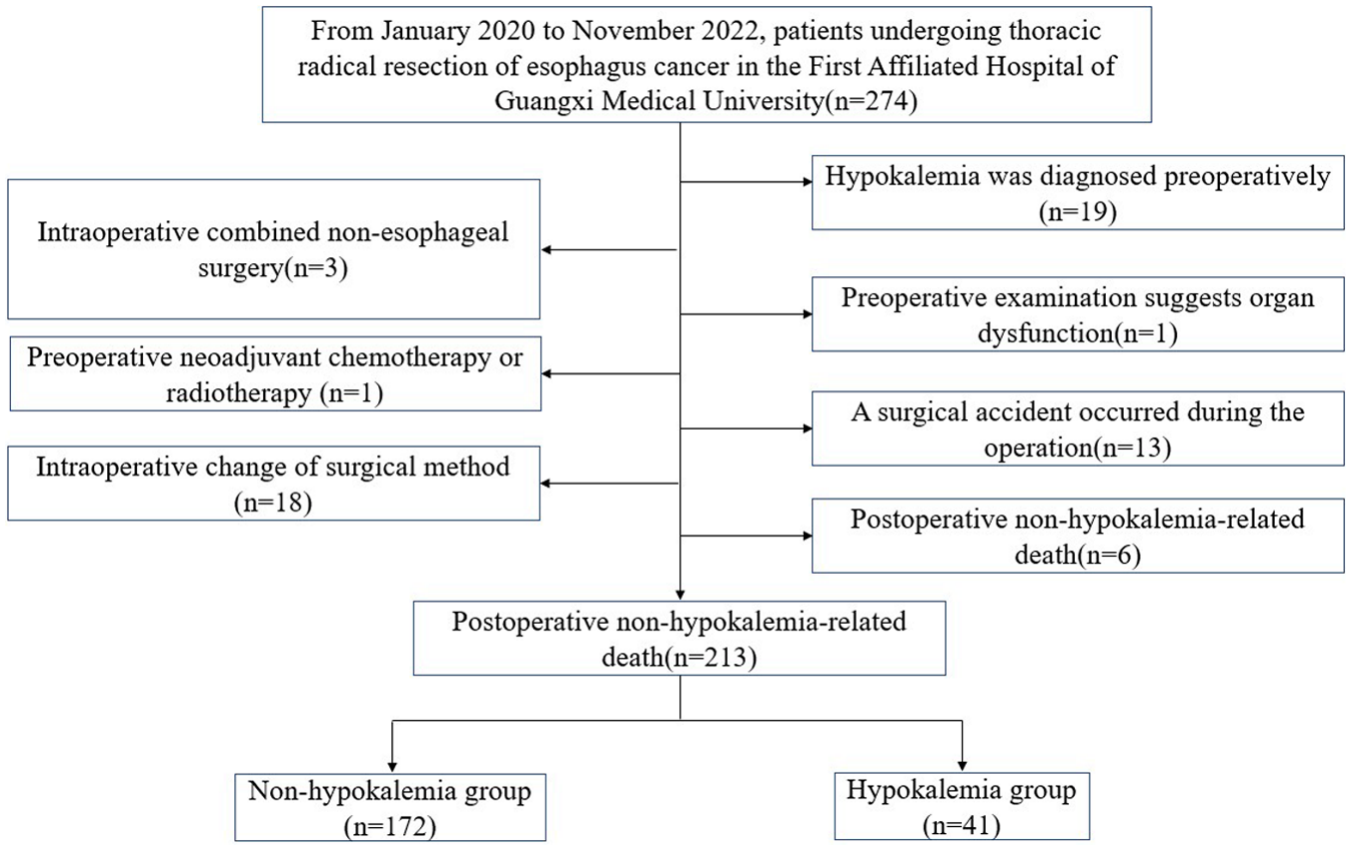
Decision curve analysis (DCA) plot.

In order to further evaluate the model, a calibration curve was also plotted, and the results showed that the predicted value of the nomogram risk prediction model was consistent with the actual observed value, with a C-index of 0.758 (0.70 < C-index ≤ 0.90 indicates that the model has a good predictive value) (Figure 4). To solve the problem that the ROC curve cannot help judge the actual utility of the clinical model, a decision curve analysis (DCA) was conducted. DCA is a simple mathematical model that formulates intervention measures based on the probability of adverse events to evaluate the availability and effectiveness of the prediction model, improving patient health and promoting the clinical application of personalized treatment. In the DCA curve, the abscissa is the threshold probability of MPE, and the ordinate is the net benefit rate or the proportion of benefited patients (Figure 5). Assuming that all patients do not suffer from hypokalemia and do not receive treatment, the net benefit rate is 0 (bottom horizontal line marked “None”); if all patients develop hypokalemia, the patient's net benefit is as the area under the backslash marked “All.” When the threshold probability was between 0.05 and 0.95, the patients had a high clinical net benefit rate; when the threshold probability was 0.45, the patients had the highest clinical net benefit.

## 4 Discussion

China has the highest EC morbidity and mortality rate globally, accounting for more than 50% of the world's incidence. Surgery is still the first choice for early EC treatment (11). Studies have reported potential risk factors for hypokalemia-related complications after gastrointestinal surgery, including surgical methods and living habits (12, 13). Herein, the differences in various indicators were analyzed between the two groups according to whether hypokalemia occurred in EC patients after the operation, and white blood cell count, serum albumin level, direct bilirubin, and operation time were risk factors. Then, a predictive model was constructed to assess the patient's risk of developing postoperative hypokalemia.

Recent research into the pathophysiological mechanisms underlying postoperative hypokalemia has yielded novel insights into its complexity. In a gastrointestinal tumor surgery study, Liang et al. found a negative correlation between surgical trauma size and hypokalemia incidence. Also, the hypokalemia incidence and related complications were significantly lower in patients receiving laparoscopic surgery than those with open surgery (14). Zhu et al. also considered that the suppression of gastrointestinal motility caused by surgery is a high-risk factor for perioperative hypokalemia (15). The application of diuretics aggravates potassium loss by enhancing urinary excretion (16). Moreover, the stress response induced by surgery increases the secretion of adrenaline and cortisol, facilitating the intracellular translocation of potassium and resulting in a decrease in serum potassium levels (17). Additionally, magnesium deficiency significantly impacts potassium metabolism by impeding the cellular uptake and transport of potassium, thus exacerbating hypokalemia (18). However, most of the above studies were based on gastrointestinal tumors rather than EC. Therefore, whether different surgical methods in EC patients are related to hypokalemia occurrence remains unclear. In the present study, EC patients who underwent open surgery experienced significantly less postoperative hypokalemia than those who underwent minimally invasive surgery. According to existing theories, compared with minimally invasive surgery, the operator has better operating space and vision when performing open surgery and can fully repair the wound and stop bleeding, reducing potassium loss in the blood (19). At the same time, patients undergoing open surgery generally have a longer recovery time of gastrointestinal function, receiving longer and stricter parenteral nutrition, which is different from patients undergoing minimally invasive surgery who receive early enteral nutrition after surgery (20). In the analysis of postoperative blood test indicators, the total protein, albumin, and calcium ions in patients with postoperative hypokalemia were lower than those without hypokalemia. Moreover, hypokalemia often causes pathophysiological changes in the kidneys, resulting in decreased tubular reabsorption and disturbance of acid-base balance, which might lead to increased loss of proteins and other ions through the kidney (21). On the other hand, the gastrointestinal dysfunction caused by hypokalemia reduces the patient's electrolyte and protein intake, aggravating the disturbance of potassium ion metabolism (22).

In this study, one phenomenon should be noted: patients with postoperative hypokalemia had lower red blood cell counts and hemoglobin concentrations, and the operation time was significantly longer than those in the control group. Due to the possible difficult operation or insufficient intraoperative hemostasis during treatment, some patients might have developed hemorrhagic anemia after the operation, and the concentration of potassium ions and hemoglobin decreased with the loss of red blood cells (23). Therefore, clinicians should check the wound carefully during the operation to stop the bleeding in time and adequately. Additionally, the surgical indications of patients need to be carefully evaluated. Open surgery should be selected for patients with difficult surgery or prone to insufficient hemostasis to avoid hypokalemia.

Previous studies have suggested a mutually reinforcing relationship between inflammatory responses and hypokalemia. After surgery, the inflammatory response in the patient's body causes cells to release cytokines and interleukins to modulate the immune response, enhancing renal tubular excretion of potassium and leading to hypokalemia (24). Conversely, releasing hormones such as aldosterone and norepinephrine when the body's potassium concentration is low increases the risk of an inflammatory response (25). Interestingly, the present study found an increased hypokalemia risk in patients after EC resection when the white blood cell count was normal. However, based on the existing research conclusions, the body might produce a stress response after surgery (especially open surgery), causing the muscles to release many potassium ions into the blood, improving the body's hypokalemia. Also, there is a relationship, a certain positive correlation, between the inflammatory and stress responses (26, 27). Therefore, open surgery may be an effective preventive measure for patients who may develop postoperative hypokalemia. However, whether there is a relationship between the size of surgical trauma and hypokalemia still needs further clarification.

This study excluded esophageal cancer patients who received neoadjuvant therapy to avoid potential confounding effects on postoperative hypokalemia. Neoadjuvant therapies, such as chemotherapy and radiotherapy, may alter electrolyte metabolism, gastrointestinal function, and renal function, thus influencing the mechanisms underlying postoperative hypokalemia (35894276). Therefore, excluding these patients allowed for a focused examination of the impact of surgery alone. However, this study also has some limitations. First, this was a retrospective, single-center study with incomplete data collection. Second, because some patients did not follow the doctor's advice to return to the hospital for reexamination after discharge, their prognosis information could not be collected. At the same time, non-standard nutritional support during diagnosis and treatment, including nutrient ratio, treatment course, and route, might affect the above results. Finally, the sample size of patients included was too small to conduct intervention studies on EC subgroups, limiting the prediction model's applicability. Future studies should adopt prospective designs, multi-center approaches, larger sample sizes, and extended follow-up periods to validate these findings and improve their generalizability. Furthermore, including patients undergoing neoadjuvant therapy and conducting stratified analyses to identify risk factors across various treatment settings would provide valuable insights.

## 5 Conclusion

The prediction model for hypokalemia risk using individualized scores on the patient's white blood cell count, serum albumin level, direct bilirubin, and operation time can screen out high-risk patients who might develop hypokalemia, which is beneficial to clinical practice. This model might also comprise a novel reference for doctors focused on screening and follow-up of patients and for the formulation of preoperative and postoperative intervention strategies.

## Data Availability

The original contributions presented in the study are included in the article/Supplementary Material, further inquiries can be directed to the corresponding author.
